# Leflunomide Reduces Proliferation and Induces Apoptosis in Neuroblastoma Cells *In Vitro* and *In Vivo*

**DOI:** 10.1371/journal.pone.0071555

**Published:** 2013-08-09

**Authors:** Shunqin Zhu, Xiaomin Yan, Zhonghuai Xiang, Han-Fei Ding, Hongjuan Cui

**Affiliations:** 1 State Key Laboratory of Silkworm Genome Biology, Southwest University, Chongqing, China; 2 College of Life Science, Southwest University, Chongqing, China; 3 Cancer Center and Department of Pathology, Georgia Health Sciences University, Augusta, Georgia, United States of America; Univ of Bradford, United Kingdom

## Abstract

Leflunomide as an immunosuppressive drug is generally used in the treatment of rheumatoid arthritis. It inhibits DHODH (dihydroorotate dehydrogenase ), which is one of the essential enzymes in the *de novo* pyrimidine biosynthetic pathway. Here we showed that leflunomide significantly reduced cell proliferation and self-renewal activity. Annexin V-FITC/PI staining assay revealed that leflunomide induced S-phase cell cycle arrest, and promoted cell apoptosis. *In vivo* xenograft study in SCID mice showed that leflunomide inhibited tumor growth and development. We also observed that DHODH was commonly expressed in neuroblastoma. When treated with leflunomide, the neuroblastoma cell lines BE(2)-C, SK-N-DZ, and SK-N-F1 showed dramatic inhibition of DHODH at mRNA and protein levels. Considering the favorable toxicity profile and the successful clinical experience with leflunomide in rheumatoid arthritis, this drug represents a potential new candidate for targeted therapy in neuroblastoma.

## Introduction

Neuroblastoma (NB) is a common childhood malignant tumor of neural crest origin, arising in the paravertebral sympathetic ganglia and the adrenal medulla [1]. The clinical characteristics of neuroblastoma are heterogeneity, metastasis and high malignancy [2], resulting in lower survival rates in patients. During the past 15 years, treatment of NB has included high-dose chemotherapy accompanied by autologous stem cell transplantation [3]. Once the NB recurrence the possibility survival of patients was very small [4].

As the fourth enzyme of the pyrimidine synthesis pathway, DHODH can oxidize dihydroorotate to orotate. This is an essential biological process which would be a potential drug target in cancer treatment [5], [6]. Inhibition of DHODH activity would reduce some essential pyrimidine nucleotides [7].

Leflunomide is characterized as an anti-inflammatory and immunomodulatory drug which was introduced for the treatment of rheumatoid arthritis (RA) in 1998 [8]. Leflunomide can inhibit pyrimidine nucleotide synthesis through directly blocking the activity of DHODH [9]–[13]. In additional to treat the rheumatoid arthritis, leflunomide is also used as a drug against the cytomegalovirus and the BK viruses [14]–[18]. Some reports have shown that DHODH inhibition through leflunomide was effective for treatment of some cancers including gliomastoma, and breast cancer [19]–[21]. DHODH inhibition led to a remarkable decrease in melanoma growth both in the zebrafish and mouse model [22]. In this study, we showed that DHODH was commonly expressed in neuroblastoma. Leflunomide treatment in neuroblastoma showed a great inhibition of DHODH expression and tumor growth when administered in clinically reasonable concentrations. Therefore, leflunomide may be a viable treatment option for neuroblastoma.

## Methods

### Cell Culture

Human neuroblastoma cell lines SK-N-F1, and SK-N-DZ were grown in Dulbecco’s modified Eagle’s medium (DMEM) supplemented with 10% fetal bovine serum (FBS) and 1% antibiotics penicillin and streptomycin (P/S); BE(2)-C was cultured in a 1∶1 mixture of Dulbecco’s modified Eagle’s medium and Ham’s nutrient mixture F12, supplemented with 10% fetal bovine serum (FBS) and 1% antibiotics (P/S). All three cell lines were purchased from ATCC. The growth media, antibiotics and FBS were purchased from Invitrogen. Leflunomide (Sigma, L5025) was dissolved in DMSO. All cells were cultured at 37°C in a 5% CO_2_ humidified incubator.

### Cell Growth Assay

The CCK-8 growth assay protocol was used as recommended by the manufacturer (Beyotime, China). Briefly, about 1000 cells in 200 µL medium were seeded in 96-well plates and incubated with leflunomide at 12.5 µM, 25 µM, 50 µM, 100 µM, and 200 µM concentration, DMSO was used as a control. 20 µL CCK-8 was added to each well and incubated at 37°C for 2 hours, the absorbance was measured at a wavelength of 450 nm.

### Cell Cycle Assay

Cells were plated in 10 cm plates and treated with 100 µM leflunomide. After 72 hours of treatment, cells were fixed with 70% ethanol, stained with propidium iodide (PI), and analyzed by flow cytometry. The data were analyzed with CellQuest Pro software (BD BioSciences). The experiments were repeated at least three times.

### Apoptosis Assay

The apoptotic ratios of cells were determined with the Annexin V-FITC apoptosis detection kit (Sigma). Briefly, after 72 hours leflunomide treatment, the cells were collected and washed twice with cold PBS buffer, resuspended in 100 µL of binding buffer, incubated with 5 µL of Annexin V conjugated to FITC and 10 µL PI for 15 min at room temperature, and analyzed by flow cytometry. Cells treated with DMSO were used as the negative control.

### Real-time PCR

Cells were treated with 100 µM leflunomide for 72 hours and harvested. Total RNA was extracted by Trizol method and reverse transcribed into cDNA by M-MLV (Promega). The DHODH mRNA transcripts were determined using the SYBR Green PCR Master Mix (Takara) by Real-time PCR. The individual values were normalized to that of the GAPDH control, and the ratio of the relative expression levels over that of the vehicle-treated cells was calculated.

### Immunofluorescence

Cells were grown on coverslips and treated with either DMSO or 100 µM leflunomide. After 72 hours treatment, the thymidine analog BrdU (5-bromo-2-deoxyuridine; Sigma) stock solution at 10 mg/ml in saline was diluted 1000× in the culture medium and incubated for 30 min. Cells were washed with PBS, fixed for 20 min in 4% paraformaldehyde (PFA), and permeabilized with 0.3% Triton X-100 for 5 min. The cells were blocked with 10% goat serum in PBS for 1 h, incubated with a primary rat antibody against BrdU (1∶200, ab6326, Abcam) in blocking buffer for 1 h at room temperature, and then incubated with the secondary antibody Alexa Fluor 488 goat anti-rat IgG (H+L) 1∶800. Incubation with 300 nM DAPI in PBS for 5 min was used for counterstaining. Cells were examined using a Nikon microscope (80i) with Image-Pro Plus software for image analysis, and calculating BrdU uptake in 10 microscopic fields.

### Western Blotting Assay

Cells treated with leflunomide for 72 hours were harvested and suspended in RIPA Lysis Buffer (Beyotime). Protein concentrations were determined with Enhanced BCA protein assay kit (Beyotime), and bovine serum albumin was used as reference. Fifty micrograms of protein was separated on 10% sodium dodecyl sulfate polyacrylamide gels (SDS-PAGE), transferred to PVDF membranes, and probed with monoclonal antibodies against DHODH (Santa Cruz, sc-166377) or α-tubulin(Cell Signaling Technology, #3873), respectively. Horseradish peroxidase-conjugated goat anti-mouse (Santa Cruz, sc-2005) was used as the secondary antibody. Proteins were visualized with BeyoECL Plus (Beyotime, China).

### *In vivo* Tumorigenic Assay

BE(2)-C cells were grown to 70–80% confluence and trypsinized. 1×10^6^ cells in 200 µL DMEM were injected into the flanks of SCID mice. After 2 weeks of tumor growth, the mice were administered intraperitoneal injections of leflunomide at 7.5 mg/kg daily or vehicle control DMSO for 12 days. Tumor diameter were measured with digital calipers every three days, and tumor volume was calculated by 4/3πr^3^. After treatment, mice were sacrificed by CO_2_, and tumors were observed and weighed. All animal experiments were pre-approved by the Institutional Animal Care and Use Committee of the Southwest University.

### Quantification and Statistic Analysis

Quantitative data are expressed as the mean ± SD. The two-tailed Student’s *t-*test was performed for paired samples. A minimum of three independent experiments were performed. Differences were considered statistically significant at *p*<0.05.

## Results

### 1. Leflunomide Inhibited Cell Growth in Neuroblastoma

**Figure 1:**
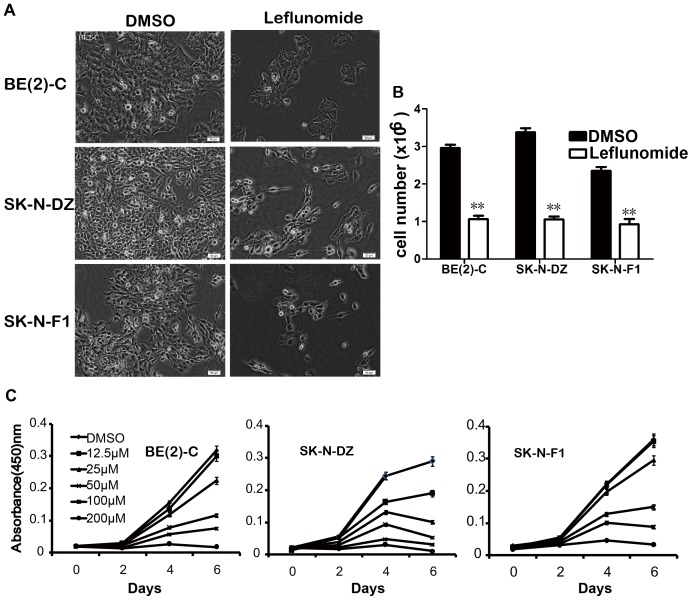
Leflunomide inhibits cell growth in neuroblastoma. A. Morphologic examination of neuroblastoma cell lines either untreated or treated with leflunomide 100 µM for 72 hours respectively. *Scale bar*, 50 µm. B. The cell counting of BE(2)-C, SK-N-DZ, and SK-N-F1 cells after 100 µM leflunomide treatment for 72 hours, DMSO as control. Data represent the average ± SD of at least three independent experiments. **, p<0.001. C. BE(2)-C, SK-N-DZ, and SK-N-F1 all three cell lines were treated with 12.5, 25, 50, 100, and 200 µM leflunomide, DMSO was used as a control. Cell growth was tested by the CCK-8 assay every 2 days. Data represent the average ± SD of at least three independent experiments.

We treated three neuroblastoma cell lines with 100 µM leflunomide for 72 hours, the result showed leflunomide can dramatically inhibit cell growth, which promoted a concomitant decrease in cell number compared with the DMSO-treated controls either in BE(2)-C, SK-N-DZ, or SK-N-F1(Figure 1A). More than 60% cell number reduction was observed in all three cell lines compared with the DMSO-treated control (Figure 1B).

To further investigate the cytostatic effects of leflunomide, cell growth rate was determined by the CCK-8 assay. The result showed that leflunomide inhibited cell growth in a time and dose-dependent manner (Figure 1C). Leflunomide at 100 µM concentrations seemed to be preferred in triggering cell proliferation inhibition, so 100 µM leflunomide was used in following experiments.

### 2. Leflunomide Reduced Cell Proliferation in Neuroblastoma

**Figure 2:**
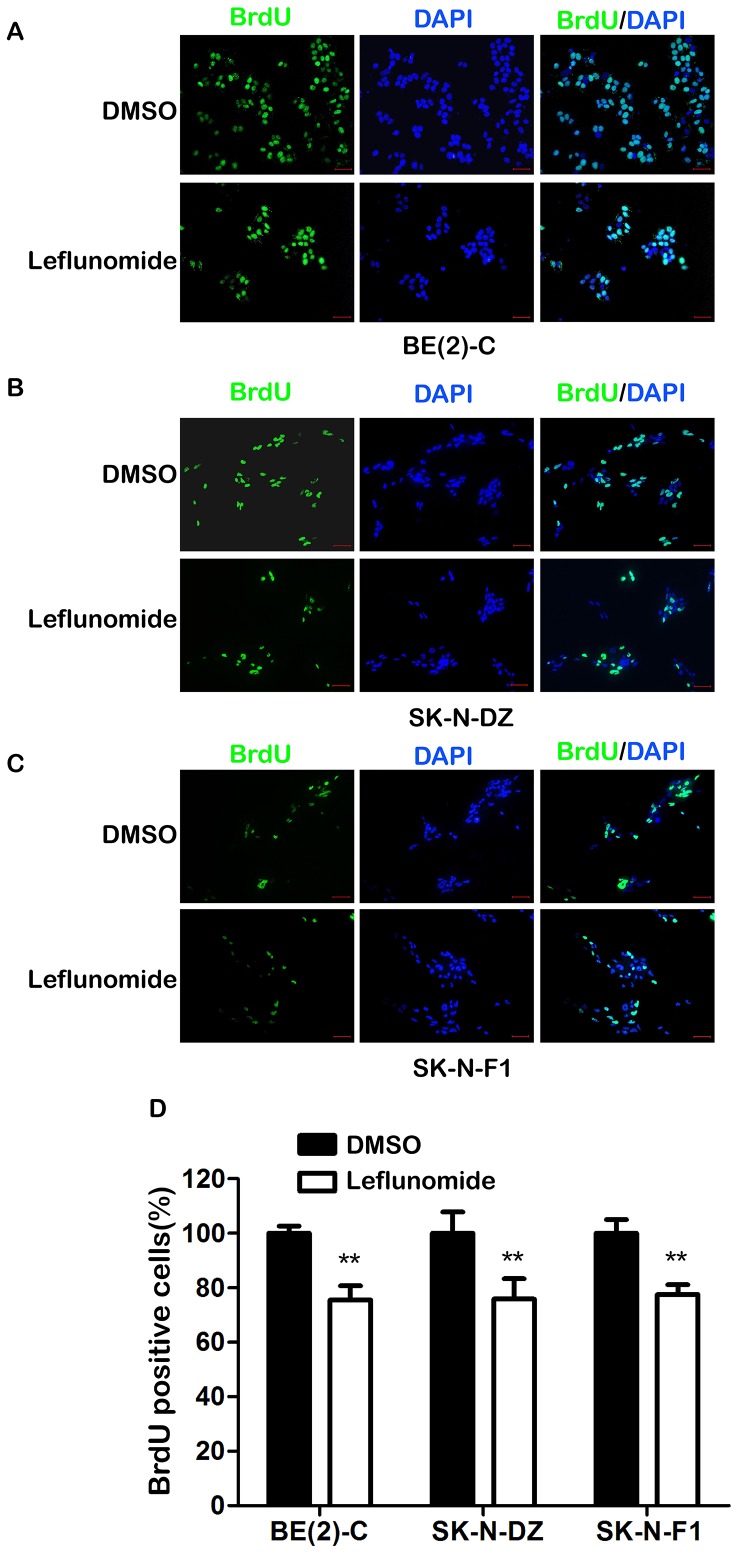
Leflunomide inhibits cell proliferation in neuroblastoma. A–C. BE(2)-C, SK-N-DZ, SK-N-F1 cells were grown on coverslips and treated with either DMSO or 100 µM leflunomide for 72 hours respectively. Cells were stained with an antibody against BrdU (green), counterstained with DAPI (blue), *scale bars,* 100 µm. D. Calculated the percent of BrdU+ cells by 20× field view of Microscope. Data represent the average ± SD of at least three independent experiments. Statistical analysis was performed using the 2-tailed Student *t*-test. **, p<0.01.

Next we employed BrdU immunofluorescence staining assays to detection the DNA synthesis analysis [23], [24]. After treated with leflunomide for 72 hours, the BrdU-positive cells significantly decreased in either BE(2)-C, SK-N-DZ, or SK-N-F1 cells compared with DMSO-treatment groups (Figure 2A–C).The statistical analysis revealed that leflunomide induced a significant reduction in the percentage of BrdU-positive cell (Figure 2D).

**Figure 3:**
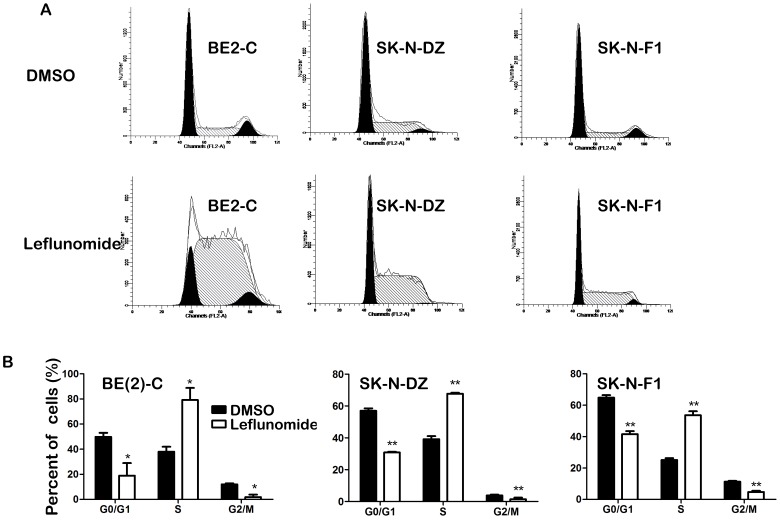
Leflunomide induces S phase cell cycle arrest in neuroblastoma. A. Neuroblastoma cells BE(2)-C, SK-N-DZ, and SK-N-F1 were treated with leflunomide for 72 hours. Cells were harvested, fixed with ethanol and stained with propidium iodide, DNA content was determined by flow cytometry. B. The statistical analysis of three neuroblastoma cell lines percentage of cell cycle phase. Data represent the average ± SD of at least three independent experiments. Statistical analysis was performed using the 2-tailed Student *t*-test. **, p<0.01.

As illustrated in Figure 3A, 100 µM leflunomide also caused an accumulation in S phase arrest of all the BE(2)-C, SK-N-DZ, and SK-N-F1 cells. The statistical analysis revealed that three of them did undergo cell cycle arrest after leflunomide treatment (Figure 3B). The percentage of S phase of BE(2)-C increased from 38.06% to 79.17%, SK-N-DZ from 39.19% to 67.71%, SK-N-F1 from 15.17% to 53.62% respectively. In summary, these observations showed that leflunomide inhibited cell proliferation through cell cycle arrest.

### 3. Leflunomide Induced Cell Apoptosis in Neuroblastoma

**Figure 4:**
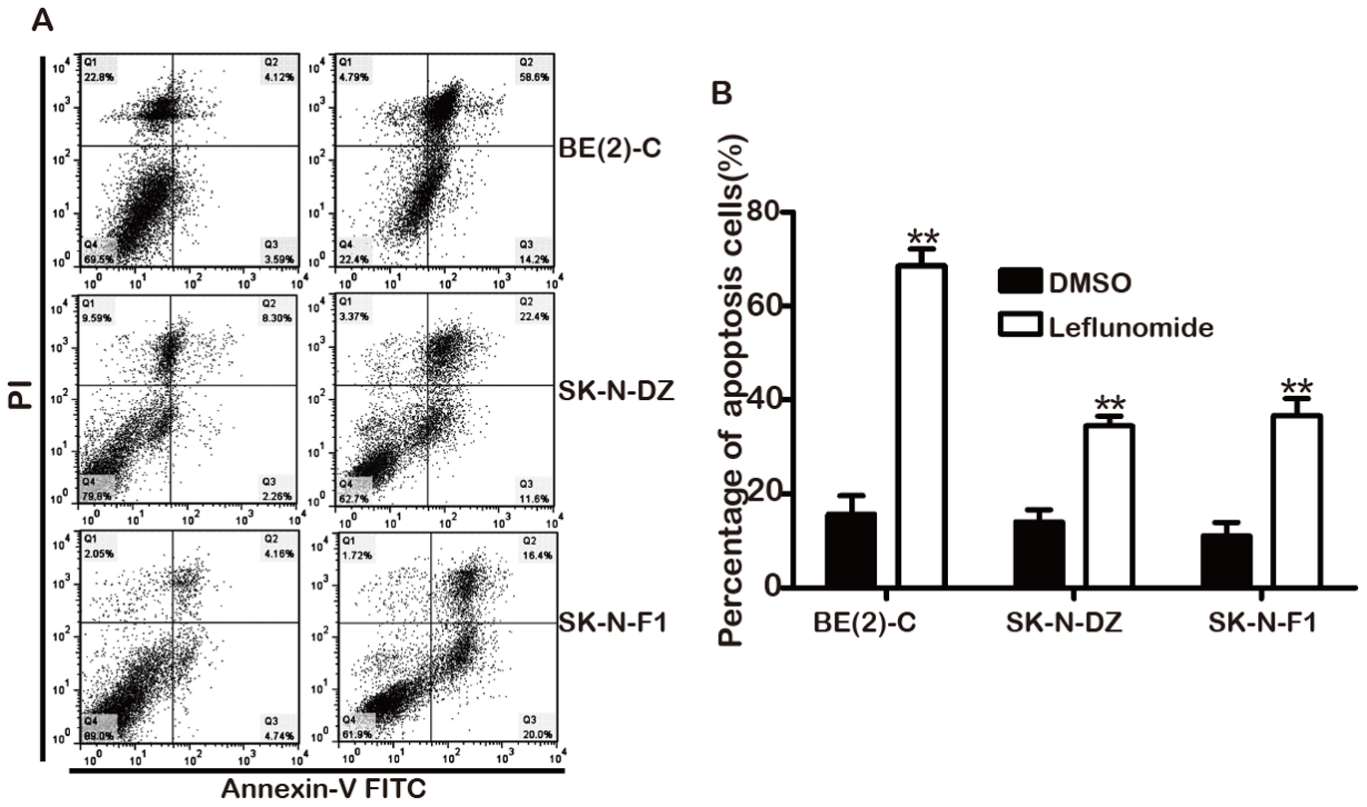
Leflunomide induces apoptosis in neuroblastoma. A. BE(2)-C, SK-N-DZ, and SK-N-F1 neuroblastoma cells were treated with 100 µM leflunomide for 72 hours, and induction of apoptosis was determined using Annexin V FITC/propidium iodide staining. B. The statistical analysis of three representative neuroblastoma cell lines percentage of apoptosis. Data represent the average ± SD of at least three independent experiments. Statistical analysis was performed using the 2-tailed Student *t*-test. **, p<0.01.

After 72 hours leflunomide treatment, cell apoptosis was accessed by Annexin V-FITC/PI staining assay. The results demonstrated that the apoptotic cells was increased dramatically in BE(2)-C, SK-N-DZ, and SK-N-F1 all three cell lines compared with the DMSO-treated groups(Figure 4A). The percentage of apoptotic value of BE(2)-C raised from 15.7% to 68.9%, SK-N-DZ from 14.1% to 34.4%, and SK-N-F1 from 11.1% to 36.6%(Figure 4B). These data suggested that leflunomide inhibited neuroblastoma cell growth through accelerating apoptosis.

### 4. Leflunomide Blocked Tumor Growth in Mouse Xenograft Model of Neuroblastoma

**Figure 5:**
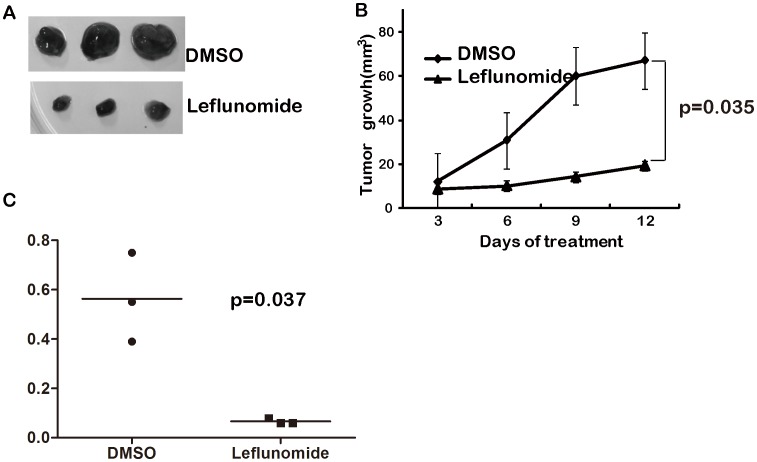
Leflunomide represses tumor growth *in vivo*. A. The NOD/SCID mice xenograft tumor after treated by DMSO or leflunomide. B. Xenograft tumor volume were analyzed per group. Data were analyzed with 2-tailed Student *t* -test with the P value indicated. C. Scatter plot of xenograft tumor weight with horizontal lines indicating the mean value. Data were analyzed with 2-tailed Student *t*- test with the *P* value indicated.

To evaluate the role of leflunomide in the development of neuroblastoma in vivo, 1×10^6^ BE(2)-C cells were injected into the NOD/SCID mice.After 2 weeks tumor growth, leflunomide was used for 12 days treatment. The result showed that the leflunomide-treated group led to nearly 70% regression in tumor size compared with the DMSO-treated group (Figure 5A–B), so did the tumor weight of leflunomide- treated group (Figure 5C). These data demonstrated that leflunomide induced tumor regression either in volume or weight. We also kept track of the mice body weight and there was no significant change during the leflunomide treatment (Data not shown). It implied no apparent side effect on the growth of mice. These results suggested that leflunomide could inhibit neuroblastoma tumor growth *in vivo*. It may be a promising candidate drug for neuroblastoma treatment.

### 5. Leflunomide Inhibited Cell Proliferation and Induced Cell Apoptosis through Down-regulation of DHODH in Neuroblastoma

**Figure 6:**
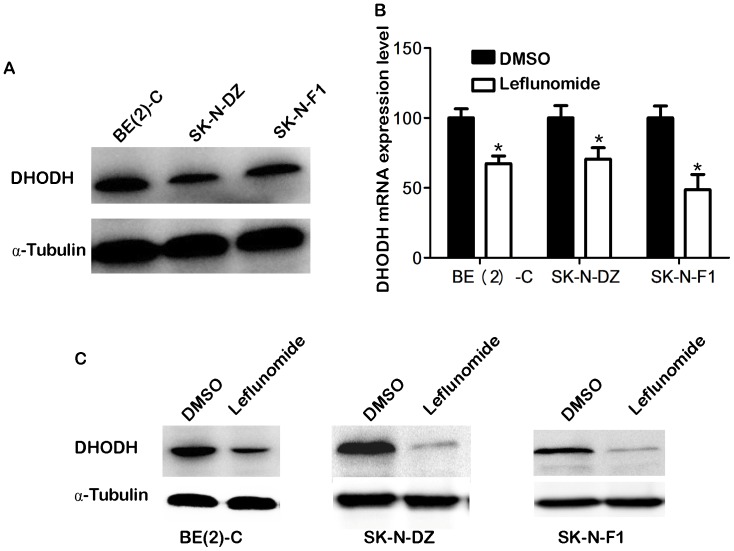
Leflunomide inhibited the DHODH expression in neuroblastoma. A. DHODH was commonly expressed in neuroblastomal cell lines.The neuroblastoma cell lines BE(2)-C, SK-N-DZ, and SK-N-F1 were harvested and performed Western blotting assay. α-tubulin was used as a loading control. B.The DHODH Relative mRNA levels were determined by RT-PCR in three neuroblastoma cell lines treated with either DMSO or 100 µM leflunomide for 72 hours. Data represent the average ± SD of at least three independent experiments. Statistical analysis was performed using the 2-tailed.Student *t*-test. *, p<0.05. C.The DHODH protein expression levels were detected by western blotting in three neuroblastoma cell lines treated with either DMSO or 100 µM leflunomide for 72 hours. α-tubulin as the control.

We employed Western blotting assay to detect DHODH protein expression levels. We found that DHODH was commonly expressed in BE-2C, SK-N-DZ, and SK-N-F1 all three neuroblastoma cell lines (Figure 6A). It means that the DHODH pathway is activated in neuroblastoma cells.

We performed quantitative reverse-transcription PCR (qRT-PCR) and western blotting to detect the DHODH expression level after 72 hours leflunomide treatment. The data showed that leflunomide can dramatically reduce DHODH expression at either mRNA (Figure 6B) or protein level (Figure 6C). All the three neuroblastoma cell lines reduced DHODH protein expression significantly after leflunomide incubation. These results indicated that leflunomide inhibited the proliferation and induced apoptosis through down-regulation of DHODH in neuroblastoma.

## Discussion

Leflunomide can promote cytostasis by G0/G1-phase [25], [26] or S-phase [20], [27] cell cycle arrest, and induce apoptosis [20], [25] in hematopoietic cells including normal mitogen-stimulated human T lymphocytes [26] [13], normal human mast cells [28], human chronic lymphocytic leukemia cells [27], murine leukemia cells [11], and human myeloma cells [25]. However, no data was reported that leflunomide may act as a potential cytostatic agent in neuroblastoma cells. In our study, we observed that leflunomide inhibited cell proliferation through cell cycle arrest and induced apoptosis and abolished the tumor growth of neuroblastoma.

Previous studies have shown that leflunomide induced apoptosis in p53-defective CLL cells, suggesting a p53-independent mechanism of apoptosis induction by leflunomide [29], with similar results reported in multiple myeloma cells [25]. Our studies showed that leflunomide significantly induced apoptosis in neuroblastoma cells. However, in most of neuroblastoma, p53 is wild type and active. The apoptosis induced by leflunomide in neuroblastoma is p53-dependent or not remains unknown and we would test it in our future study.

Some reports revealed that leflunomide suppressed *de novo* pyrimidine synthesis and some essential mitochondrial function, which lead to cell cycle arrest and cytostasis in the premalignant and malignant prostate epithelial cells [30]. We observed a dose-dependent cytotoxic effect for leflunomide in the neuroblastoma treatment. There is no report about DHODH expression in neuroblastoma. We found that DHODH was common expressed in all three neuroblastoma cell lines. The expression of DHODH was significantly decreased either at mRNA or protein level after leflunomide treatment. In our future study, we will focus on the DHODH function in neuroblastoma.

Since leflunomide has been used in treatment of rheumatoid arthritis for a long period, there are considerable human data available regarding clinical tests and adverse drug reactions [31]. Our study revealed that leflunomide inhibited cell proliferation and tumor growth through down-regulation DHODH pathway in neuroblastoma cells. Leflunomide could represent a promising new drug candidate for neuroblastoma treatment.
